# Evaluation of the cardioprotective effects of crystalloid del Nido cardioplegia solution via a rapid and accurate cardiac marker: heart-type fatty acid-binding protein

**DOI:** 10.3906/sag-2002-53

**Published:** 2020-06-23

**Authors:** Mehmet KİRİŞCİ, Aydemir KOÇARSLAN, Duygun ALTINTAŞ AYKAN, Filiz ALKAN BAYLAN, Adem DOĞANER, Yavuz ORAK

**Affiliations:** 1 Department of Cardiovascular Surgery, Faculty of Medicine, Kahramanmaraş Sütçü İmam University, Kahramanmaraş Turkey; 2 Department of Pharmacology, Faculty of Medicine, Kahramanmaraş Sütçü İmam University, Kahramanmaraş Turkey; 3 Department of Biochemistry, Faculty of Medicine, Kahramanmaraş Sütçü İmam University, Kahramanmaraş Turkey; 4 Department of Biostatistics, Faculty of Medicine, Kahramanmaraş Sütçü İmam University, Kahramanmaraş Turkey; 5 Department of Anesthesiology and Reanimation, Faculty of Medicine, Kahramanmaraş Sütçü İmam University, Kahramanmaraş Turkey

**Keywords:** Heart-type fatty acid-binding protein, cold blood cardioplegia solution, del Nido solution, coroner artery bypass grafting, myocardial protection

## Abstract

**Background/aim:**

Our aim in this study was to compare the efficacy and safety of crystalloid del Nido solution and cold blood cardioplegia solution on clinical and laboratory parameters.

**Materials and methods:**

Sixty patients who underwent elective coronary bypass operation between July 2019 and January 2020 were included in our study. Patients were divided into 2 groups of 30 patients using del Nido solution (DNS) and cold blood cardioplegia solution (CBCS), which were given for cardiac arrest. Demographic data, preoperative, postoperative 0th h, 6th h and 4th day creatine kinase myocardial band (CK-MB) and troponin I values were compared with a specific cardiac enzyme heart-type fatty acid-binding protein (H-FABP).

**Results:**

We found that aortic cross clamp duration and cardiopulmonary bypass (CPB) time were shorter in patients using del Nido solution than cold blood cardioplegia solution (57.30 ± 23.57 min, 76.07 ± 27.18 min, P = 0.006) (95.07 ± 23.06 min, 114.13 ± 33.93, P = 0.014). Total cardioplegia solution volume was higher in the cold blood cardioplegia solution group (1426.67 ± 416.00 vs. 1200 ± 310.73 P = 0.02). Preoperative and postoperative levels of cardiac enzymes including CK-MB, troponin I and H-FABP were comparable in del Nido solution and cold blood cardioplegia solution groups.

**Conclusion:**

According to these results, when we compare both demographic data and CK-MB, troponin I and H-FABP levels, both cardioplegia solutions were comparable regarding safety and efficacy in terms of myocardial protection.

## 1. Introduction

Myocardial damage during cardiac surgery is the most important cause of mortality and morbidity. Cardioplegia has been used for many years in cardiac surgery for myocardial protection due to systemic and local hypothermia [1,2]. Cardioplegia solution, which is the technique of providing elective and chemically cardiac arrest, was first applied in 1955 by Melrose with potassium [3]. As open heart surgery develops structurally and physiologically, it has led to the development of new types of cardioplegia solution. In 1990, Dr. del Nido and researchers at the University of Pittsburg developed a cardioplegia solution, a mixture of long-acting blood and crystalloids, especially for pediatric patients. Changes have been made in the original solution, and is currently known as “del Nido cardioplegia solution” in the clinical practice [4]. The addition of polarizing agents such as lidocaine is thought to reduce the energy consumption, and the presence of calcium-competing ions such as magnesium at optimum concentration reduce intracellular calcium concentration, thereby preventing the cell damage [5]. In the literature, there are many studies showing the safety and efficacy of adult cardiac surgery with del Nido cardioplegia solution [6–11].

Heart-type fatty acid binding protein (H-FABP) is a small cytoplasmic protein (15 kDa) and is expressed 2–10 times more in the cardiac muscle than in the skeletal muscle. Its physiological functions are the protection of cells during ischemia through the transport of fatty acids, the synthesis of membranes and mediators, gene expressions, and inactivation of the radicals [12–14]. Following myocardial damage, it enters the circulation rapidly and reaches high concentrations in the serum within 1 h after myocardial injury [15,16]. H-FABP has been accepted as an early marker for acute myocardial infarction (MI), but is not yet common in clinical use [17]. Cardiac troponins are specific markers for MI and are mainly released from the necrotic cardiac myocytes [18]. H-FABP is also released from the necrotic cardiac myocytes, but unlike troponin, it is released from the ischemic cardiac myocytes as well [19–21].

Our aim in this study was to compare the serum H-FABP, creatine kinase myocardial band (CK-MB) and troponin I levels following the administration of crystalloid del Nido solution and the traditional cold blood cardioplegia solution for cardiac arrest during coronary bypass operation. 

## 2.Materials and methods

### 2.1. Patients

The study was approved by the Institutional Ethics Committee (approval date: July 03, 2019, document number: 3). Before participation in the study, informed written consent was obtained from each subject after explaining the protocol.

A total of 60 patients who underwent elective coronary artery bypass operation in the Cardiovascular Surgery Department of Kahramanmaraş Sütçü İmam University Research Hospital between July 2019 and January 2020 were included in the study. They were randomly divided into 2 groups of 30 patients. The first group was composed of patients using del Nido cardioplegia solution (group DNS) for cardiac arrest during the operation, and the second group was composed of patients with traditional cold blood cardioplegia solution (group CBCS). All patients were operated with cardiopulmonary bypass (CPB) under general anesthesia. The patients with reoperation due to bleeding, acute MI, congenital heart disease, cardiomyopathy, valvular heart disease, congestive heart failure, malignancy, infectious diseases, acute or chronic inflammatory diseases, psychiatric diseases, neurological diseases, hematological diseases, renal failure, liver failure and hematological diseases were excluded from the study.

### 2.2. Anesthesia technique

Standard anesthesia at therapeutic doses was applied to all patients. Radial artery catheterization was performed and monitored. Induction of anesthesia was achieved with midazolam (Zolamid®, Defarma, Tekirdağ, Turkey, 0.1 mg/kg, intravenously), fentanyl (Talinat®, Vem, İstanbul, Turkey, 5–8 mcg/kg, intravenously) and rocuronium bromide (Myocron®, Vem, 0.6 mg/kg, intravenously). General anesthesia was achieved with anesthesia device (Drager, Lubeck, Germany), and anesthetic agent 2 % MAC (Minimum alveolar concentration) Sevoflurane (Sevorane®, Abbvie, İstanbul, Turkey). Rocuronium bromide (0.6 mg/kg, intravenously) was administered every 30 min.

### 2.3. Surgical technique

All surgical procedures were performed in mild hypothermia (32 oC), accompanied by aortaatrial cannulation, with CPB, with the help of nonpulsatile ruller pump, membrane oxygenator and standard extracorporeal circuits. To keep the activated clotting time (ACT) value above 480 s, 320–400 IU/kg unfractionated heparin was administered intravenously. 1350 mL prime volume (1 L isolate S, 20% mannitol 200 cc, 3% NaCl 150 cc, 5000 IU heparin) were taken into the pump lines. During the cardiopulmonary pump, 2.0–2.5 L/min/m² flow rate, mean arterial pressure 60–80 mmHg, 180–200 mm Hg PaO2 and 35–45 mmHg PCO2 were maintained. Body temperature adjustment was cooled at a rate of 1 °C per min and was performed by reheating at a rate of 1 °C every 3 min. Antegrade cardioplegia cannula was placed in the ascending aorta. Following aortic clamp, 1000 mL del Nido cardioplegia solution was given once at +4 °C. An additional dose was administered if the del Nido cardioplegia solution exceeded 60 min. 

Conventional cold blood cardioplegia solution was given as 1000 mL at 32 °C. In the ischemic period, an additional 500 mL dose was administered every 20 min. Before opening the aortic cross clamp (ACC), approximately 500 mL of warm blood (hot shot; 37 °C) was applied through an antegrade cardioplegia catheter to facilitate spontaneous heart contraction. Proximal anastomoses were performed with a side clamp on the ascending aorta. After the CPB was over and the cannulas were removed, the unfractionated heparin was neutralized with protamine.

Del Nido cardioplegia solution and the traditional cold blood cardioplegia solution contents were shown in Table 1.

**Table 1:** The components of the solution used in the del Nido and cold blood cardioplegia.

Variables components	DNS	CBCS
Basic solution	İsolayt S* 1 L	Blood, 1 L
KCL (mEq/L)	26	22.5 for induction 7 to remain
NaHCO3 (mEq/L)	13	10
% 20 Mannitol (mL)	17	20
% 15 MgSO4 (mL)	14	10
% 2 Lidocaine (mL)	6,50	0
Blood/crystalloid rate	1/4	4/1

DNS: Del Nido solution, CBCS: Cold blood cardioplegia solution, * İsolayt S (Na:141mEq, K: 5 mEq, Mg: 3 mEq, Cl: 98 mEq, Asetat: 27 mEq, Fosfat: 1 mEq, Glukonat: 23 mEq, pH 7.40), NaHCO3: Sodium bicarbonate, KCL: Potassium chloride, MgSO4: Magnesium sulfate.

### 2.4. Laboratory measurements

Cardiac troponin I and CK-MB values were measured in preoperative, postoperative 0th h, 6th h and 5th day blood samples. In addition, preoperative, postoperative 6th h and postoperative 5th day blood samples were taken to search for H-FABP values. Serum and plasma were separated for H-FABP examination and stored at −80 °C for analysis. Serum CK-MB and troponin I levels were studied on Roche Cobas 8000 automatic analyzer. Results for CK-MB and troponin I were determined as μg/L. 

Serum H-FABP levels (Bioassay Technology Laboratory, Shanghai, China) were studied using the ELISA (enzyme-linked immunosurbent assay) method, automatic ELISA reader (Thermo Fisher Scientific, Vantaa, Finland) and computer program (Scanlt for Multiscan FC 2.5.1). For H-FABP: Sensitivity: 0.01 μg/mL, Assay range: 0.05 μg/mL–20 μg/mL, intraassay coefficient of variation (CV) % <8%, and interassay % CV <10%. The results were determined as μg/ml.

### 2.5. Statistical analysis

The suitability of the variables to normal distribution was examined with the Shapiro-Wilk test. Comparisons between groups were evaluated with the independent samples t test. Covariance Analysis (ANCOVA) was applied to compare group differences in postoperative measurements independently of preoperative differences. Frequency distribution differences between categorical variables were performed with chi-square test and Fisher’s exact test. Statistical significance was accepted as P < 0.05. Data was evaluated in IBM SPSS version 22.0.

## 3. Results

The demographic data of the patients were given in table 2. There was no difference in demographic characteristics between the 2 groups. All intraoperative data and postoperative clinical results were presented in Table 3. Aortic cross clamp (ACC) time and CPB time was shorter in patients using del Nido cardioplegia solution (group DNS) than in the group using cold blood cardioplegia solution (group CBCS) (57.30 ± 23.57 minutes, 76.07 ± 27.18 min, P = 0.006 and 95.07 ± 23.06 min, 114.13 ± 33.93, P = 0.014). The total volume of cardioplegia solution used was 1200 ± 310.73 mL in the group using DNS and 1426.67 ± 416.00 mL in the group using CBCS, and there was a significant difference between the groups (P = 0.02). The number of grafts with coronary bypass was 3.57 ± 0.90 in group DNS, 3.77 ± 0.86 in group CBCS and there was no significant difference between groups (P = 0.383). Postoperative atrial fibrillation (AF) was developed in nine patients in Group DNS, and 5 patients in the group CBCS. When 2 groups were compared, there was no statistically significant difference between the 2 groups, although more patients developed postoperative AF in group DNS (P = 0.222). Postoperative low cardiac output syndrome (LCOS) was developed in 2 patients in group DNS, and in 1 patient in group CBCS, there was no statistically significant difference (P = 0.554). In-hospital mortality was developed in 2 patients in Group DNS, and 1 patient in group CBCS, and there was no statistically significant difference between groups in terms of mortality (P = 0.554). There was no significant difference between groups in developing postoperative renal failure (P = 0.554). When intraoperative defibrillation requirements were compared, there was no significant difference (P = 0.640). There was no significant difference between the groups in terms of intensive care stay and hospital stay (P = 1.000 and P = 0.524, respectively). Preoperative and postoperative (0. h, 6. h and 5. day) CK-MB and troponin I levels, and preoperative and postoperative (6. h and 5. day) H-FABP results were presented in Table 4. When the 2 groups were compared, there was no statistically significant difference in CK-MB, troponin I and H-FABP values.

**Table 2:** The baseline features of the patients included in the present study.

	Group DNS (n = 30)	Group CBCS (n = 30)	P value
Sex (Female/male)	10/20	8/22	0.57
BMI, kg/m2	29.82 ± 3.02	30.15 ± 3.11	0.68
Age, years	59.03 ± 11.58	62.00 ± 11.53	0.62
Diabetes mellitus	12 (40.00%)	14 (46.67%)	0.60
Hypertension	14 (46.67%)	13 (43.33%)	0.80
Hyperlipidemia	10 (33.33%)	12 (40.00%)	0.59
Peripheral artery disease	6 (20.00%)	8 (26.67%)	0.54
Chronic renal failure	0 (0.00)	0 (0.00)	-
Chronic obstructive pulmonary diseases	5 (16.67%)	6 (20.00%)	0.74
Smoking	8 (26.67%)	9 (30.00%)	0.77
Ejection fraction	52.33 ± 8.38	52.97 ± 10.00	0.79

Independent samples t test; Chi-Square test; Fisher’s exact test; α: 0.05; DNS: Del Nido solution; CBCS: Cold blood cardioplegia solution; BMI: Body mass index; SD: Standart deviation. Note: Values are presented as mean ± SD and percentage.

**Table 3:** The intraoperative data and postoperative outcomes.

	Group DNS	Group CBCS	P value
Bypass number	3.57 ± 0.90	3.77 ± 0.86	0.38
Cross time, min	57.30 ± 23.57	76.07 ± 27.18	0.01*
Cardiopulmonary bypass time, min	95.07 ± 23.06	114.13 ± 33.93	0.01*
Total cardioplegia volume, mL	1200.00 ± 310.73	1426.67 ± 416.00	0.02*
Postoperative renal failure	2 (6.67%)	1 (3.33%)	0.55
Postoperative atrial fibrillation	9 (30.00%)	5 (16.67%)	0.22
Mortality	2 (6.67%)	1 (3.33%)	0.55
Low cardiac output syndrome	2 (6.67%)	1 (3.33%)	0.55
Intensive care stay time, days	2.23 ± 0.68	2.23 ± 0.43	1.00
Hospitalization time, days	7.11 ± 0.63	7.00 ± 0.64	0.52
Intraoperative defibrilation	3 (10.00%)	2(6.67%)	0.64

Independent samples t test; Chi-Square test; Fisher’s exact test; α: 0.05; DNS: Del Nido solution; CBCS: Cold blood cardioplegia solution. Note: Values are presented as mean ± SD and percentage.

**Table 4:** The mean levels of preoperative and postoperative cardiac enzymes and H-FABP.

		Group DNS	Group CBCS	P value
H-FABP (μg/ml)	Preoperative	5.49 ± 1.46	5.54 ± 1.51	0.90a
	Postoperative 6th hy	6.47 ± 1.65	6.23 ± 1.13	0.29b
	Postoperative 4th dayz	6.52 ± 1.72	6.44 ± 1.57	0.89b
CK-MB (ng/mL)	Preoperative	2.30 ± 0.87	2.33 ± 0.75	0.90a
	Postoperative 0th hx	28.37 ±7.46	28.83 ± 8.04	0.92b
	Postoperative 6th hy	31.83 ± 5.99	32.47 ± 6.10	0.70b
	Postoperative 4th day z	3.28 ± 1.45	3.38 ± 1.34	0.87b
Troponin I (ng/mL)	Preoperative	0.04 ± 0.03	0.04 ± 0.02	0.63a
	Postoperative 0th hx	3.12 ± 1.24	3.24 ± 1.17	0.71b
	Postoperative 6th hy	5.91 ± 1.81	5.83 ± 1.63	0.95b
	Postoperative 4th dayz	0.65 ± 0.35	0.66 ± 0.27	0.99b

aIndependent samples t test; bCovariance analysis (ANCOVA); α: 0.05; x(Dependent variable: Postoperative 0th h, Covariate: Preoperative); y(Dependent variable: Postoperative 6th h, Covariate: Preoperative, postoperative 0th h); z(Dependent variable: Postoperative 4th day, Covariate: Preoperative, postoperative 0th h, postoperative 6th h); DNS: Del Nido solution; CBCS: Cold blood cardioplegia solution; H-FABP: Heart-type fatty acid-binding protein; CK-MB: Creatine kinase-MB. Note: Values are presented as mean ± SD.

## 4. Discussion

This study was performed to investigate the effectiveness of 2 different cardioplegia solutions used for cardiac arrest during coronary bypass surgery. We evaluated the myocardial protection by measuring the cardiac enzymes CK-MB and troponin I, compared to H-FABP, which is sensitive and fast-releasing biomarker for myocardial damage. Both crystalloid-based or blood-based, all methods of cardioplegia work for hyperkalemic electromechanical diastolic arrest. Our aim was to provide a clear still vision in our surgical field, and to protect the myocardium from ischemic damage as much as possible.

In our study, patients were divided into 2 groups each consisted of 30 patients according to the solutions used for cardiac arrest: the group using CBCS and the group using DNS. When these 2 groups were compared, there was no difference in terms of demographic data. The absence of significant differences in demographic data between the 2 groups indicates that the groups were homogenized. This enables us to obtain more accurate results in order to compare the heart-protective effect of 2 cardioplegia methods in our study. Del Nido cardioplegia solution is repeated every 60 min, it may be repeated once every 90 min in several clinics [22,23]. We found that cross clamp time and CPB time in del Nido cardioplegia solution group were lower than the group using cold blood cardioplegia solution. We think that this is due to the differences in administration intervals, since del Nido cardioplegia solution was given once every 60 min, while cold blood cardioplegia solution was given once every 20 min. Administration of cold blood cardioplegia solution every 20 min increases the volume of cardioplegia solution. In the study performed by Ucak et al., in patients who underwent coronary bypass by using del Nido cardioplegia solution and blood cardioplegia solution, the cross clamp time and total CPB durations were shorter, and total volume of cardioplegia solution was less in del Nido cardioplegia solution, similar to our study [24]. In our study, there was no statistically significant difference between the groups in terms of intraoperative defibrillation requirement, LCOS, postoperative AF, postoperative kidney failure, intensive care stay and hospitalisation. In a meta-analysis performed by Guru et al., they showed that patients with blood cardioplegia solution had lower postoperative LCOS and lower mean postoperative CK-MB values compared with patients using crystalloid cardioplegia solution [25]. In another meta-analysis performed by Sa et al., they showed that there was no significant difference between patients using crystalloid cardioplegia solution and those with blood cardioplegia solution in terms of developing LCOS [26].

Increased CK-MB and troponin I values in the postoperative period estimate the myocardial damage in patients undergoing cardiac arrest with CPB [27]. In our study, there were no difference in CK-MB and troponin I levels between the groups, which were examined consecutively. Jonge et al. showed in their 12-year retrospective research that the postoperative mean CK-MB value of patients using blood cardioplegia solution was significantly higher than that of crystalloid cardioplegia solution [28]. Ucak et al examined postoperative CK-MB and troponin I levels in isolated coronary bypass operations using del Nido solution and blood cardioplegia solution. They showed that there was no significant difference between the 2 groups between the mean CK-MB and troponin I levels [24]. In the meta-analysis conducted by Li et al., over a number of 836 patients using del Nido cardioplegia solution and traditional blood cardioplegia solution were compared. They showed that there was no difference between the 2 groups in terms of postoperative mortality, development of atrial fibrillation (AF), levels of CK-MB, troponin I and troponin T [29]. 

H-FABP is placed in the cytoplasm of striated muscle cells and is rapidly released in response to myocardial damage [30]. Upon myocardial damage, H-FABP is quickly released from myocytes into the systemic circulation due to its small size and free cytoplasmic localization. It has been suggested that transient increases in sarcolemmal membrane permeability allowed H-FABP escape into the systemic circulation. Previous studies claimed that myocyte damage may occur even after short-term ventricular stress [31,32]. H-FABP has a potential role in cardiomyocyte differentiation. Its relationship with decreased cell proliferation in the mice cardiomyocytes have been demonstrated by in vivo and in vitro studies [33,34]. Wang et al. indicated that overexpression of H-FABP inhibited cell proliferation in bone marrow-derived mesenchymal stem cells [35]. In addition, Zhu et al. demonstrated that P19 embryonic myocardial cell line that overexpresses H-FABP inhibited cell proliferation and promoted apoptosis during myocardial cell development [36]. In ventricular myocytes of newborn rats under hypoxia, down regulation of H-FABP suppressed cell apoptosis and improved structural remodeling. Besides, H-FABP upregulation has been shown to increase the phosphorylation in mitogen activated protein kinases (MAPK) signaling pathway and decrease phosphorylated protein kinase B (Akt) levels, thus, leading an increase in apoptosis and in remodeling [37]. Elimination of H-FABP occurs through the kidney, hence, creates a diagnostic window for patients with normal kidney function [38].

In previous studies, H-FABP has been shown to function as a reliable diagnostic marker in patients with acute coronary syndrome, peripheral vascular ischemia, cardiac surgery, and coronary artery surgery [39–42]. Our study is the first assay in the literature to compare the effect of 2 different cardioplegia solutions on myocardial protection by meausring serum H-FABP levels. H-FABP acts as a transporter of myocardial fatty acids, and is released into the circulation immediately after myocardial damage. H-FABP is a more sensitive biomarker that shows a much faster increase than CK-MB and troponin I, the biomarkers showing myocardial damage after coronary bypass [43,44]. Thielman et al. evaluated the troponin I and H-FABP levels at 1st, 6th, 12th, 24th, 48th, and 72th h postoperatively to determine the myocardial damage in patients undergoing coronary bypass. They showed that the rapid increase in serum H-FABP values is a more specific biomarker in determining the perioperative myocardial damage [44]. Jo et al. showed that H-FABP was more sensitive than CK-MB and troponin T to postulate the myocardial damage in patients undergoing cardiac surgery with CPB [45]. Assuming that H-FABP is superior to conventional cardiac biomarkers to predict early perioperative myocardial damage after coronary artery bypass graft (CABG) surgery, we analyzed the serum levels of H-FABP at preoperative, postoperative 6th h and 4th day to compare the effect of 2 different cardioplegia solution. According to our findings, the mean H-FABP value did not differ significantly in all time periods using both cardioplegia solutions. Hence, both cardioplegia solutions we used seem reliable in terms of myocardial protection due to CK-MB, troponin I and H-FABP levels.

In conclusion, our clinical and laboratory results were very satisfactory using DNS in patients undergoing CABG surgery. The results of our study showed that both del Nido cardioplegia solution and traditional cold blood cardioplegia solution that we routinely used in our clinics were effective and usable. In many respects, both methods are close to each other and do not provide any obvious superiority. In addition to the common cardiac enzymes CK-MB and troponin I, H-FABP, which is a more sensitive enzyme against cardiac ischemia, showed that 2 cardioplegia methods were both safe and effective. This study is a single-center study focusing on the clinical results of a limited number of adult coronary surgical cases. It is not possible to reveal a general conclusion based solely on these results. Further studies on myocardial cells, especially at the molecular level, would provide more informative data on the importance of the use of cardioplegic methods in the future. Our results are encouraging for the further studies.

## Disclaimers/Conflict of interest

The authors declared no conflicts of interest with respect to the authorship and/or publication of this article, all authors have read and approved of the manuscript being submitted. The authors received no financial support for the research and/or authorship of this article.

## Informed consent

The study was approved by the Institutional Ethics Committee (approval date: July 03, 2019, document number: 3). Before participation in the study, all participants provided informed consent in the format required by the relevant authorities and/or boards.
